# Marine reserve effects on fishery profit

**DOI:** 10.1111/j.1461-0248.2007.01151.x

**Published:** 2008-04

**Authors:** Crow White, Bruce E Kendall, Steven Gaines, David A Siegel, Christopher Costello

**Affiliations:** 1 Department of Ecology, Evolution and Marine Biology, University of California Santa Barbara, CA 93106, USA; 2 Donald Bren School of Environmental Science and Management, University of California Santa Barbara, CA 93106, USA; 3 Marine Science Institute, University of California Santa Barbara, CA 93106, USA; 4 Institute for Computational Earth System Science, University of California Santa Barbara, CA 93106, USA

**Keywords:** Bioeconomics, density dependence, fishery profit, marine reserves, stock effect

## Abstract

Some studies suggest that fishery yields can be higher with reserves than under conventional management. However, the economic performance of fisheries depends on economic profit, not fish yield. The predictions of higher yields with reserves rely on intensive fishing pressures between reserves; the exorbitant costs of harvesting low-density populations erode profits. We incorporated this effect into a bioeconomic model to evaluate the economic performance of reserve-based management. Our results indicate that reserves can still benefit fisheries, even those targeting species that are expensive to harvest. However, in contrast to studies focused on yield, only a moderate proportion of the coast in reserves (with moderate harvest pressures outside reserves) is required to maximize profit. Furthermore, reserve area and harvest intensity can be traded off with little impact on profits, allowing for management flexibility while still providing higher profit than attainable under conventional management.

*Ecology Letters* (2008) 11: 370–379

## Introduction

Ecologists have dedicated considerable effort towards identifying management strategies that maximize fishery yields. These studies often conclude that no-take marine reserves, initially developed as a conservation tool, may also benefit some fisheries (Gerber *et al.* 2003 and references therein). Accumulation and maintenance of mature fish stocks to carrying capacity levels within reserves may enable them to serve as sources to adjacent fishing areas through spill-over of adults and export of larvae across reserve boundaries (Crowder *et al.* 2000; Kellner *et al.* 2007). Such benefits of reserves to fisheries may outweigh costs of prohibiting fisheries from directly utilizing the protected areas, producing higher yields than those sustainable under conventional, quota-based management (Halpern & Warner 2003).

Polacheck (1990) predicted spawning stock biomass to increase within reserves, but for this to rarely lead to optimal levels of spill-over that increase fishery yield. Elegant analytical analyses by Hastings & Botsford (1999) demonstrated that exploitation by fisheries of larval export from reserves can generate equivalent yields compared with those under conventional management; this result was elaborated upon by others who found reserves to increase yield, especially for species with intercohort post-dispersal density dependence (Neubert 2003; Gaylord *et al.* 2005; White & Kendall 2007). For these species, harvesting of adult populations between reserves reduced their competitive and predatory effects on younger conspecific individuals, thereby increasing the recruitment rate of larvae exported from reserves into fished areas. Intercohort post-dispersal density dependence is common among nearshore fish (Hixon & Webster 2002), especially among predatory species (Hallacher & Roberts 1985; Hobson *et al.* 2001), who also happen to be priority target species for many fisheries (CDFG 2004).

In the above studies, all proposed strategies that increase fishery yield by creating reserves rely on fishing pressures in unprotected areas that are more intense than the optimal fishing pressure under conventional management. However, from the perspective of fisheries, net economic profit, not gross yield, is the appropriate metric for measuring the welfare of their industry and appreciation for alternative management policies, which often fail when lacking the endorsement of and cooperation from local fisheries (Crutchfield 1973; Kaplan 1998; Davis 2005). Furthermore, because the cost to catch a fish increases as the density of a fish population declines (the ‘stock effect’, Clark 1990), a strategy that relies on more intensive fishing pressures may compromise profits: increased yields with reserves may not generate higher profits, and reserve management that only equals yields attainable under conventional management will reduce profits.

Using a generalized bioeconomic model affected by environmental stochasticity, Armsworth & Roughgarden (2003) demonstrated that a system may increase its economic value when it incorporates reserves that promote ecological stability. Stefansson & Rosenberg (2005, 2006) constructed discrete-time bulk biomass models with nested economic fleet models that incorporated stochasticity in the form of management uncertainty; they too found reserves to buffer negative economic effects introduced by stochasticity. These studies support the notion that reserve-based management represents a precautionary approach that may increase the overall stability of fish populations and fishery yields, thus the economic value of the fishery, in the face of environmental variability and uncertainty in knowledge of ecological conditions critical to effective management (Lauck *et al.* 1998; Carr & Raimondi 1999).

In one of the few papers to explicitly consider the stock effect under a broad range of fish population and fishery harvest conditions, Sanchirico *et al.* (2006) analysed a bioeconomic model with spatial heterogeneity in economic and ecological parameters, and identified conditions when reserve-based management maximized long-term profit to fisheries. Their model is deterministic, and includes interpatch larval dispersal and density-dependent recruitment. Among other less intuitive conditions, Sanchirico *et al.* determined a patch to be a likely candidate for closure if it was characterized by low biological productivity, high harvest cost and net exportation of biomass. Under no conditions did they find reserve-based management to be optimal when habitat quality was homogeneous and interpatch dispersal was symmetrical. However, their model had interpatch dispersal between only two patches of equal size, thus the only reserve strategy possible was exactly 50% closure of the coastline, and the fish density outside the reserve is spatially uniform (there is no possibility for increased densities at the boundaries of reserves).

Here, we analyse a model that is similar in biological and economic assumptions to Sanchirico *et al*., but introduce space as a continuous, linear coastline and assume that dispersal is localized. We ask if reserve-based management strategies predicted to increase fishery yield can also increase fishery profit, despite their reliance on increased harvest pressures compared with conventional management. Our results are a striking contrast to the conclusions of Sanchirico *et al.*

## Methods

We focused our analysis on nearshore fish and invertebrate species characterized by a sessile adult stage subject to density-independent mortality, and a pelagic larval stage that disperses. We developed an integrodifference model with these simple elements of life history:
(1)
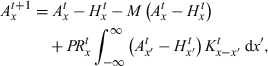

where *t*, *x* and *x′* refer to time and two locations along a uniform coastline, respectively, *A* = number of adult fish (units arbitrary), *H* = harvest, *M* = natural annual adult mortality rate, *P* = adult per capita production of larvae that survive to settlement, *K*_*x − x′*_ = the proportion of larva settling at location *x* that originated from location *x′* and *R* = ecruitment probability of settling larvae. The larval dispersal kernel represented by *K*_*x − x′*_ is Gaussian, based on simulations of ocean mixing processes, and adjustable via a chosen mean larval dispersal distance *D*_d_ (Siegel *et al.* 2003; and calculations therein). The model is discretised into 1-year time steps and 1-km length segments along the coast, and fish mature at 1 year. Harvesting occurs after dispersal but before the next bout of reproduction. Thus, fishing mortality begins when fish are *c.* 1 year old. This model is identical to the single-size-class model presented by Gaylord *et al.* (2005, p. 2181; eqn 1), with the following differences: the coastline is not limited in length, inclusion of *H* allows for non-zero fishery escapement levels, and we focused solely on intercohort post-dispersal density dependence. Recruitment success of larvae decreases exponentially with increasing adult abundance at the settlement location:
(2)
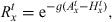

where *g* characterizes the severity of the density-dependent recruitment process. Equation 1 represents a single adult stage class preceded by a single pre-recruit stage class. This conservative approach reduces unnecessary complexity (White & Kendall 2007), as well as enables eqn 2 to capture density-dependent recruitment processes occurring shortly after settlement (e.g. via depredation, Hobson *et al.* 2001), during the juvenile/subadult stage (e.g. via competition and/or territoriality, Larson 1980; Love *et al.* 1991) or both.

We limited fish and invertebrate movement to their larval stage during dispersal. Although spill-over caused by adult movement may affect benefits of reserves to fisheries (Kellner *et al.* 2007), we assumed along-shore site fidelity, because it is common in many nearshore fish and invertebrates (e.g. Topping *et al.* 2005).

**Figure 1:**
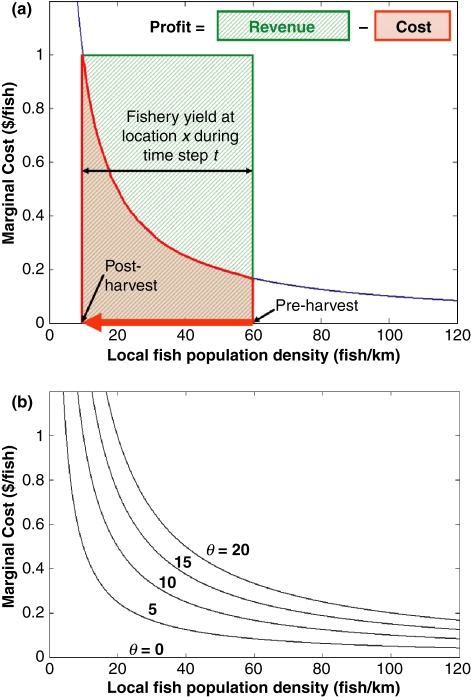
Stock effect curve(s) estimating marginal cost relative to local fish population density. (a) Schematic representation of the calculation of fishery profit at one location during a single harvest season, as a function of revenue based on a fixed market price ($1) per fish unit, minus the sum of the marginal costs of harvesting down the local fish population. In this example the fishery harvested to the ‘zero marginal profit’ level, where (marginal revenue)/(marginal cost) = 1. (b) Stock effect curves representing fishery species that are of different marginal costs to harvest. Increasing values of *θ* represent fishery species that are intrinsically more expensive to harvest.

Profit to a fishery is a function of revenue gained from selling fish yield, minus the cost of catching those fish. We modelled the marginal cost of fishing to be inversely proportional to local fish density, *θ*/(fish*km^−1^) (Clark 1990), where higher values of *θ* represent species that are intrinsically more expensive to harvest. For each 1-km distance bin along the coast, the annual cost of harvesting was calculated by integrating along the stock effect curve from the pre-harvest to post-harvest population density (Fig. 1a). We then subtracted local cost from local revenue, based on a fixed market price of $1 per fish, and averaged across the entire coastline to estimate mean profit ($*km^−1^). To highlight the difference between reserve based, and conventional fisheries management, we examined sustainable profit, and thus implicitly assumed zero discounting.

Let the virgin carrying capacity be 100 fish*km^−1^ and *A* be the fish density below which marginal cost would exceed marginal revenue; because price = 1, *A* = *θ.* This ‘zero marginal profit’ point represents a (marginal revenue)/(marginal cost) rate equal to 1, and is the density below which no fisherman, no matter how myopic, would harvest. As a result, *θ* is the percentage of virgin density below which fishing would naturally cease.

When left without escapement restrictions, modern fisheries worldwide have demonstrated their ability to exploit fish populations to remarkably low escapement levels (i.e. < 20% of original stock), and maintain over many years sufficient harvest pressures for preventing recovery of stocks from those low levels (Lipcius & Stockhausen 2002; Cardinale & Svedang 2004; Worm *et al.* 2006). *θ* = 0 represents a fishery being able to harvest with perfect efficiency. We evaluated fishery management across this range of conditions (0 ≤ *θ*≤ 20, Fig. 1b).

To eliminate confounding effects caused by common pool management (where profits may be dissipated from the race to fish, Gordon 1954), we approached the problem from the perspective of several independent fisheries each having exclusive access to a particular species represented by a stock effect value. Species-specific regulatory rules of escapement and harvest distribution were implicitly enforced on each fishery by an outside agency or internally (e.g. by a fishing cooperative).

Model scenarios represented the full factorial of adult productivity, adult annual natural mortality, mean larval dispersal distance and stock effect parameter values in Table 1. Equilibrium adult density (i.e. carrying capacity) was scaled to 100 fish*km^−1^ in the absence of fishing mortality [i.e. given set demographic values, the density dependence coefficient was solved: *g* = 100^−1^log(P*M^−1^)].

**Table 1:** Symbol, value(s) and description of design parameters (*A, M, P, g, D*_d_*, θ* and *p*) and variables (escapement and proportion coast in reserves). Fish units are numerical and arbitrary. A full factorial of all values was simulated. Marginal cost equals marginal revenue when (*A*_eq_[*H* = 0])(escapement) =*θ%.* See Siegel *et al.* (2003) for calculations using *D*_d_.

Parameter/variable	Values evaluated	Description
*A*_eq_[*H* = 0]	100	Equilibrium virgin population density(fish per km), where *H* = harvest
*M*	0.05, 0.1, 0.2, 0.3	Natural annual mortality probability
*P*	1, 2, 3	Adult per capita production of larvaethat survive to settlement
*g*	=Log(P*M^−1^)/(*A*_eq_[*H* = 0])	Density-dependent recruitmentcoefficient
*D*_d_	10, 100, 200	Mean larval dispersal distance(km) for calculating *K*_*x − x′*_
*θ*	0, 5, 10, 15, 20	Stock effect coefficient ($ * km^−1^)
*p*	1	Price ($ per fish) = marginal revenue
*(A*_*x*_*− H*_*x*_*)/(A*_eq_[*H* = 0])	0.01, 0.02, 0.03… 0.9	Escapement
Frac(*x*[*H*_*x*_ = 0])	0, 0.05, 0.1,… 0.9	Proportion coast in reserves
	288 360	Total number of scenarios simulated

In each model scenario, we considered all of the 18 reserve policies listed in Table 1, including that having no reserves (i.e. conventional management). A reserve was defined as an area permanently closed to fishing. For each reserve policy, we considered a range of systematically varied reserve size and spacing configurations, from that represented by many small, closely-positioned reserves to fewer, larger reserves positioned farther apart. To maintain evenness in reserve size and spacing for each configuration, we simulated model space along a circular domain; perimeter length of the domain was adjusted (up to 1500 km) to allow for different configurations. The spatial breadth of our approach enabled us to capture the variable fish population and fishery dynamics resulting from different single large vs. several small (SLOSS) reserve configurations. However, resolving the SLOSS debate was not the goal of this study, rather, we optimized configuration to optimize reserve-based management for comparison with optimal conventional management. The homogeneous conditions of our model system were ideal for this exercise because it enabled us to explore all symmetrical reserve configurations possible within an exceptionally large coastal domain.

Given initial design variable values, and a reserve policy and configuration, we imposed each of the 89 escapement policies in Table 1 across the entire fishable domain (i.e. area between reserves). This broad range of harvest levels collectively saddles the zero marginal profit point for each value of *θ*, generating all reasonable (marginal revenue)/(marginal cost) rates, whether optimal or not.

We compared equilibrium yields (*θ* = 0) and profits (*θ* > 0) of optimal as well as sub-optimal reserve-based management strategies with those attainable under optimal conventional management. Optimal management was defined as the strategy characterized by reserve policy, configuration and escapement that maximized sustainable profit. Optimal conventional management was limited to strategies without reserves. Sub-optimal management was defined as a strategy whose maximum sustainable profit was marginally less than that achievable under optimal management.

Without reserves, all fish population and fishery dynamics in our model are spatially homogeneous; in this case identical solutions can be achieved via a constant escapement or constant effort policy (where a constant fraction of the population in each patch is harvested). With reserves, interactions among the spatially explicit protection of fish stocks, larval dispersal kernel and density dependence recruitment function generate variable fish population densities along the domain, potentially generating differences in profit attainable via policies focused on escapement vs. effort. In this study, we focused on escapement because (i) it directly related to *θ*, enabling us to most clearly illuminate how harvest pressure effects fishery profit and because (ii) regulation of escapement has in general been demonstrated to be optimal in maximizing profit from a renewable resource whose dynamics are described by either deterministic or stochastic stock-recruitment models (Reed 1979; Costello *et al.* 2001). However, there is not a consensus on the latter issue (e.g. among Walters & Parma 1996 and authors of the previous citations), and regulation of effort may better reflect practiced fishery management policies. For these reasons, we re-ran our model with harvest regulated via constant effort to determine if consideration of this policy recovered qualitatively similar results compared with those generated by the constant escapement policy.

## Results

Given a fixed fraction of the coastline in reserves, fishery yields (*θ* = 0) and profits (*θ* > 0) were maximized via similar reserve network configurations (Fig. S1, in Supplementary Material). With small or moderate proportions (e.g. < 20%) of the coast in reserves, yields and profits were similar across the full breadth of evaluated configurations. With larger proportions in reserves, yields and profits were maximized by small- or medium-sized reserves with short or moderate inter-reserve distances. We found a consistent pattern with respect to dispersal distance of the targeted fishery species: for a specified proportion of the coast in reserves, maximum or near-maximum yields and profits were generated by setting reserve width to be approximately equal to or less than the mean larval dispersal distance.

**Figure 2:**
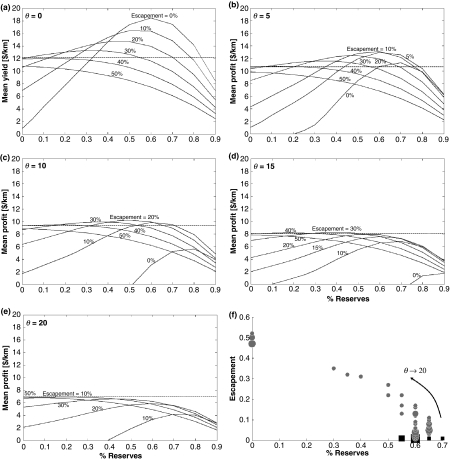
Yield and profit in relation to the stock effect, proportion of the coast in reserves and escapement in fished areas. (a–e) Yields and profits under different stock effect scenarios, given optimal configuration of reserves compromising a proportion of the coast (‘% Reserves’, where zero represents conventional management). Curved lines represent different escapement levels regulated across the fished region. For reference, the horizontal dashed lines indicate maximum yield and profits attainable under optimal conventional management. *M* = 0.1, *P* = 1, results are quantitatively identical across all evaluated mean larval dispersal distances. (f) Optimal per cent reserve and escapement policies that maximize yield (*θ* = 0, squares) and profit (*θ* > 0, circles), for all combinations of *M*, *P* and *θ* values in Table 1. In general, optimal management was characterized by decreased per cent reserves concurrent with increased escapement as *θ* increased (arrow). Symbol size corresponds with policy frequency. Conventional management (upper left points) was optimal when *P* = 1 and *θ* = 20 [e.g. panel (e)], in all of those cases, sub-optimal management with up to 35% reserves only decreased profits by < 5%.

Given our baseline life history parameters, yield under conventional management was maximized by setting escapement to 34% of virgin carrying capacity (Fig. 2a, horizontal dashed line). Maximum yields with reserves were substantially greater, but required high harvest pressure (escapement < 30%) between optimally configured reserves that constituted a large fraction (20–60%) of the coastline (Fig. 2a, curved lines). The overall maximum yield emerged when reserves constituted 60% of the coast and escapement outside them was zero. The only effect of dispersal distance was to change the ‘optimal configuration’ at each reserve proportion.

**Figure 3:**
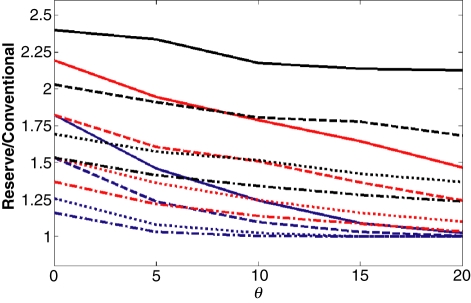
Maximum yields (*θ* = 0) and profits (*θ* > 0) under optimal reserve-based management relative to those under optimal conventional management, evaluated across fisheries targeting species represented by different stock effect scenarios. *M* = 0.05 (solid lines), 0.1 (dashed), 0.2 (dotted) and 0.3 (dash-dot), *P* = 1 (blue), 2 (red) and 3 (black).

The stock effect (*θ* > 0) reduced fishery profits under all management strategies, whether conventional or with reserves (Fig. 2b–e). Nevertheless, maximum fishery profits under optimal reserve-based management were at least approximately equal, and typically substantially greater than, those attainable under optimal conventional management, regardless of adult natural mortality (*M*) or per capita production (*P*) values (Fig. 3). We re-simulated all fish population and fishery management conditions under an effort-based regulatory policy, and found the relative increase in profits with reserves at least to equal those recovered by the escapement-based policy.

Escapement levels required to maximize profits increased with increased stock effect severity. With reserves, the optimal proportion of the coast protected from fishing also shifted (from high to low) with increasing stock effect severity (Fig. 2f). Optimal management required a particular combination of per cent reserve and escapement for each stock effect scenario. However, the stock effect actually flattens the relationship between profit and reserve area. There is a broad spectrum of similarly profitable near-optimal management strategies characterized on its extreme ends by (i) low revenue combined with low cost of fishing lightly along most of the coast and (ii) high revenue combined with high cost of fishing intensively between reserves that constituted a substantial proportion of the coast (for all strategies within this spectrum, profits exceeded maximum profit under conventional management). This spectrum allows us to design a single policy (reserve network plus escapement levels) that achieves near-optimal profit simultaneously across all values of *θ*.

**Figure 4:**
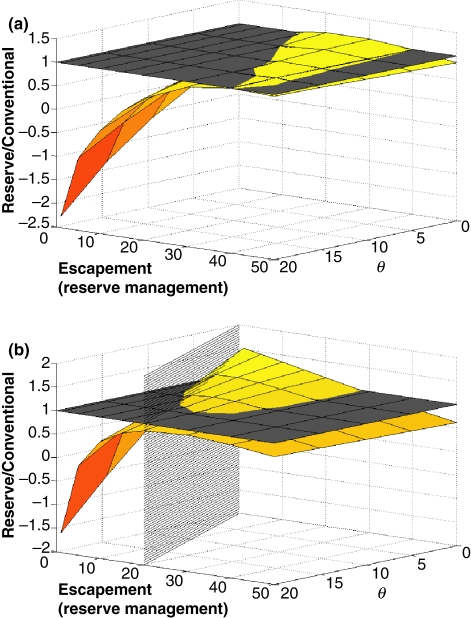
Relative difference in profit between sub-optimal reserve-based management (‘Reserve’) and optimal management without reserves (‘Conventional’). Sub-optimal reserve-based management is characterized by 20% (a) and 60% (b) of the coast dedicated to an optimally configured reserve network. Policies are evaluated across all stock effect scenarios, and regulation of escapement = 1–50%. Under optimal conventional management, escapement was set optimally to 34–47% for *θ* = 0–20, respectively. Curved surfaces represent model results, dark horizontal planes are inserted for reference, representing equivalence in profit between the two management strategies. Vertical plane is inserted in (b) for reference, representing *θ%* escapement. *M* = 0.1, *P* = 1.

As an example, management characterized by 20% reserves and optimal escapement for each *θ* generated equal or increased profits compared with optimal conventional management (Fig. 4a, along the surface ridge). Optimal escapement under this scenario varied minimally with stock effect severity (i.e. the ridge is broad and approximately orthogonal to the escapement axis). As a result, equal or increased profits compared with conventional management are obtainable across all fishery species, given regulation of single escapement level at *c.* 35%. This pattern was consistent across nearly all productivity and mortality values (Fig. S2a).

A second example, which might have greater conservation benefit, is characterized by 60% reserves and optimal escapement. This policy would at best substantially increase profits, and at least marginally decrease profits, when compared with optimal conventional management (Fig. 4b, along the surface ridge). Here, profit was more sensitive to changes in escapement than in the 20%–reserve policy presented above, and optimal escapement increased considerably with increased *θ* (i.e. compared with that in Fig. 4a, the ridge is narrower and runs at an angle with respect to the escapement axis). As a result, no single escapement level could consistently generate satisfactory profits across all stock effect conditions compared with profits attainable under conventional management. However, optimal escapement was similar to *θ%*, the zero marginal profit-escapement level (see description of θ in Methods). As a result, *θ%* escapement intersects nearly along the ridge of the profit-escapement-stock effect surface in Fig. 4b (vertical plane). Mean profit across stock effect scenarios was greater under this sub-optimal policy (60% reserves, *θ%* escapement) than under optimal conventional management, a trend that was substantially increased for nearly all fishery species (Fig. S2b).

## Discussion

Our bioeconomic model captures the fact that overly intensive harvesting is detrimental to fishery profit. As a result, and in contrast to studies focused on yield, offsetting a large proportion of the coast in reserves while allowing *c.* 0% escapement outside the reserves will not maximize profit. However, when properly accounting for the stock effect, reserves still emerged as a viable management tool under both escapement and effort-based regulation programmes. Optimal, and even moderately sub-optimal, reserve-based policies generated profits approximately equal to or greater than those attainable under optimal conventional management. The breadth of reserve-based management strategies that improve on conventional management offers the opportunity to identify solutions that can simultaneously satisfy consumptive and conservation values.

Sanchirico *et al.* (2006) also integrated the stock effect into a bioeconomic model to investigate the economic value of reserves; however, unlike that determined here, they found reserves not to be optimal when all patches are homogeneous in ecological and economic conditions. Our study reveals a previously unidentified value from reserves because we explicitly considered a coastal domain consisting of numerous patches connected by local dispersal dynamics; our approach enabled us to optimize reserve configuration, then quantify ensuing patch-specific population dynamics, associated with different per cent reserve policies. As a result, we identified optimal reserve policies (e.g. 35% reserves, given *θ* = 15, Fig. 2d) that were not even considered in Sanchirico *et al.*’s two-patch model.

Compared with that presented here, consideration of a patch-specific harvest policy within fished areas may further increase profits by enabling fisheries to generate and exploit source-sink dynamics via spatially heterogeneous harvest pressures. Recent theoretical work by Ralston & O’Farrell (in press) indicates that yield of a species with the same form of density dependence as that studied here may be maximized when spatial heterogeneity in harvest is increased to the extreme case characterized by closure of a patch. Spatial heterogeneity in harvest can also lead to reserves when patches are intrinsically heterogeneous in habitat quality, interpatch dispersal rates and/or cost of harvest (Sanchirico *et al.* 2006). However, in a bioeconomic analysis based on the California sea urchin fishery, Smith & Wilen (2003) demonstrate that overly simplistic consideration of economic incentives (e.g. proximity to port) that drive spatial heterogeneity in harvest rates by fisheries can bias results in favour of reserves.

Fishery management is fraught with uncertainty, e.g. measurement error surrounding stock assessment and regulation error surrounding enforcement of catch levels. Our model is unrealistic in assuming that the state of nature (fish abundances and parameter values) is known precisely, thus the manager is able to choose and enforce regulations exactly. However, inclusion of uncertainty in our study may not alter its qualitative conclusions in support of reserves, because consideration of uncertainty has almost always contributed substantially to the long-term sustainability advantage of reserve-based management over conventional management (see references in Introduction). Forthcoming work by Costello and Polasky (in review), provides explicit evidence in support of this concept that optimal management of a stochastic dynamic resource includes use of reserves.

The results presented here assume that the bioeconomic system is at equilibrium; in particular, fish populations within reserves are near virgin carrying capacity and therefore generating high larval export rates to fished areas (Halpern & Warner 2003). Under some conditions, high population densities may develop quickly in response to protection from fishing (Halpern & Warner 2002; but see Russ & Alcala 2004), but the short-term effects of reserves on fishable biomass levels in adjacent areas is only beginning to be revealed empirically (Lester & Halpern 2007). It is quite possible that if a fishery is currently well managed, there may be a short-term drop in profits after optimal reserves are established.

Optimal reserve configuration was characterized by reserve width being equal to or less than the mean larval dispersal distance of the targeted fishery species – a result that is supported by studies focused solely on yield (Gaylord *et al.* 2005). The same reserve design maximized both yield and profit, because it consistently (i.e. annually) simultaneously maximized yield (and thus revenue) and minimized cost of fishing. Overly large reserves were undesirable because larval export was reduced. Small reserves persisted due to inter-reserve larval recruitment; however, inordinately small reserves may not be appropriate when one considers adult movement (Halpern & Warner 2003). The symmetry of the optimal configurations presented here, due to our simplified characterization of a linear, homogeneous coast and symmetrical dispersal kernel, should not be interpreted as literal guidance for fisheries management. To the contrary, evenness in reserve configuration is likely not optimal in a spatially heterogeneous system (Kaplan 2006).

Despite long-held interest in identifying optimal management strategies that maximize yield, fisheries worldwide have rarely approached optimal yields. It is challenging and costly to enforce fishery regulations (e.g. quotas, Akiba 1997), and the data (e.g. fish stock size) required for calculating optimal regulations are often exceedingly expensive to obtain (Uozumi 2003). Given these constraints, we asked if there were sub-optimal management strategies that benefit fisheries sufficiently well compared with optimal management, while reducing costs and challenges associated with formulating and enforcing the management strategy. We explored two sub-optimal reserve-based strategies, both of which equalled or increased profit compared with conventional management, and may be more practical to implement and regulate, as well as agreeable from a non-economic perspective to fishery and conservation parties: (i) dedication of a moderate proportion (20%) of the coast to reserves concurrent with a flat escapement policy (*c.* 35%) across all species harvested and (ii) dedication of a substantial proportion (60%) of the coast to reserves with escapement outside set at the point of zero marginal profit.

Optimal management without reserves required regulation of escapement at 34–47% for *θ* = 0–20, respectively. Regulation of escapement is widespread, yet it can be challenging and expensive to enforce (Sutinen & Andersen 1985), an issue that may be exacerbated when multiple escapement levels are enforced simultaneously in a region. The first sub-optimal management strategy (Figs 4a and S2a) could ameliorate this issue by allowing managers to focus on a single escapement policy (*c.* 35%) across multiple species. A fishery targeting species that are intrinsically expensive to harvest (*θ* = 20) would equal profits compared with those under conventional management, while ones targeting less expensive species would accrue increased profits. As a result, this management strategy, representing a commitment to reserves that is already being approached in some regions (e.g. in California, CDFG 2007), may represent a viable option for simultaneously benefiting fishery and regulatory parties.

The above management policy still requires substantial effort and expense to assess stocks for calculating harvest levels, as well as enforcement of those harvest levels across each fishery. From a fishery perspective, enforcement of spatial closures represents adding yet another layer of regulation. The second sub-optimal management policy we evaluated – 60% of the coast in reserves, concurrent with *θ*% escapement across all fisheries (Figs 4b and S2b) – may circumvent this issue by redirecting management focus solely towards spatial closures. Under this policy a fishery is no longer told by regulators when it has reached its quota and must cease harvesting, a fresh change in fishery management that may be appreciated (Salas & Gaertner 2004). Instead, a fishing vessel may approach a *θ%* escapement level based on current harvest costs experienced onboard while at sea. This policy also reduces the burden of stock assessments that challenge the ability to calculate quotas. Instead, managers may focus on monitoring fishing vessel location, a task already accomplished remotely and efficiently with Global Positioning System technology (Randall 2004). Thus, in addition to the explicitly evaluated economic gains generated by this policy, the implicit costs of regulation and social conflict between the fishery and managers may be markedly reduced. However, a caveat accompanies this policy, which allows for low escapement compared with other policies we explored. High harvest pressures associated with low escapement may reduce recruitment rates in fished areas through habitat degradation (e.g. via trawling), thereby reducing the economic value of this policy.

Individual fishing vessels sometimes target multiple fishery species simultaneously, a strategy they may assert to maintain high overall catch levels amidst fluctuations in abundance of individual species (Vestergaard *et al.* 2003; Perez-Espana *et al.* 2006), or as a part of a more deterministic shift in fishing effort away from species that are less abundant (e.g. due to overfishing) and towards other species that are more abundant (Milessi *et al.* 2005). Our model does not explicitly consider multispecies fisheries; however, with some broad assumptions we can estimate qualitative effects of conventional vs. reserve-based management on such a fishery’s profit. Given exclusive access to two species (e.g. those represented by *θ* = 10 and 20), and its ability to employ a single harvest method for catching those species, a fishery is predicted to experience reduced cost per fish harvested for the more costly-to-harvest species as long as it is catching it alongside the less expensive species. In essence, the *θ* = 20 species would serve as by-catch that supplements harvest of the equal or more abundant *θ* = 10 species, which sets the cost per harvest rate for both species. Given at least equal price of the *θ* = 20 relative to the *θ* = 10 species, the reduction in cost per *θ* = 20 fish harvested would increase current-year profit gained from that species, thereby benefiting the fishery under either conventional or reserve-based management. The fishery would also have the opportunity to continue harvesting the *θ* = 20 species below its independent 20% zero marginal profit-escapement level, to a 10% zero marginal profit-escapement level set by the *θ* = 10 species, thereby further increasing profit. However, this second benefit would only be realized if sustainable management allowed for such a low escapement level. Although not guaranteed, such an option is more likely under reserve-based management, which in general allowed for higher fishing pressures in fished areas compared with those under conventional management (see Fig. 2f). The lowest escapement levels observed in our analyses occurred when reserves constituted a large proportion of the coast, suggesting that such a policy may be amenable to a multispecies fishery able to reduce the effective *θ* of secondary species that it targets.

Although our model did not explicitly evaluate effects of demographic and environmental stochasticity on fisheries management, the economic efficacy of reserves is not predicted to decrease with consideration of such stochastic processes (see Introduction). Nonetheless, there remains a paucity of studies exploring effects of stochastic processes on fishery yields and profits (Gerber *et al.* 2003), highlighting the need for future attention to this topic. We assumed several population dynamic and life history features (e.g. absence of adult growth and the form of density dependence) that could be important for our results. Consideration of the former via a age-/stage-structured model is predicted to further increase yields with reserves, because older/larger fish, which are preferentially protected in reserves, produce exponentially more offspring, resulting in greater larval export to fished areas (Gaylord *et al.* 2005). Consideration of alternative forms of density dependence may substantially alter results, especially if local adult population density no longer mediates recruitment success of settling larvae (Hastings & Botsford 1999; White & Kendall 2007). Exploration of the questions presented in this paper for fishery species exhibiting these and other life history traits is needed. In all fishery management investigations, it is important to explicitly integrate fish population dynamics with fishery economics to accurately characterize emergent bioeconomic consequences arising from alternative management strategies. Elucidation of such consequences is critical for a productive dialogue among fishery regulatory, industry and conservation parties on what strategy each considers appropriate for successful fishery management.

## Supplementary material

The following supplementary material is available for this article:

Figure S1 Effect of reserve configuration on yield and profit.

Figure S2 Change in profit to fisheries operating under suboptimal reserve-based versus optimal conventional management policies.

This material is available as part of the online article from: http://www.blackwell-synergy.com/doi/full/10.1111/j.1461-0248.2007.01151.x
