# Supplementary material for: Marine reserve effects on fishery profit
Source: Ecol Lett. 2008 Apr;11(4):370–9. doi: 10.1111/j.1461-0248.2007.01151.x (PMC2268951; doi:10.1111/j.1461-0248.2007.01151.x)
Supplement: Figure S1 — Effect of reserve configuration on yield and profit. [file ele0011-0370-SD1.doc]

**MARINE RESERVE EFFECTS ON FISHERY PROFIT**

White, C., B.E. Kendall, S. Gaines, D.A. Siegel, and C. Costello

SUPPLEMENTARY MATERIAL

**Figure S1: Effect of reserve configuration on yield and profit.**


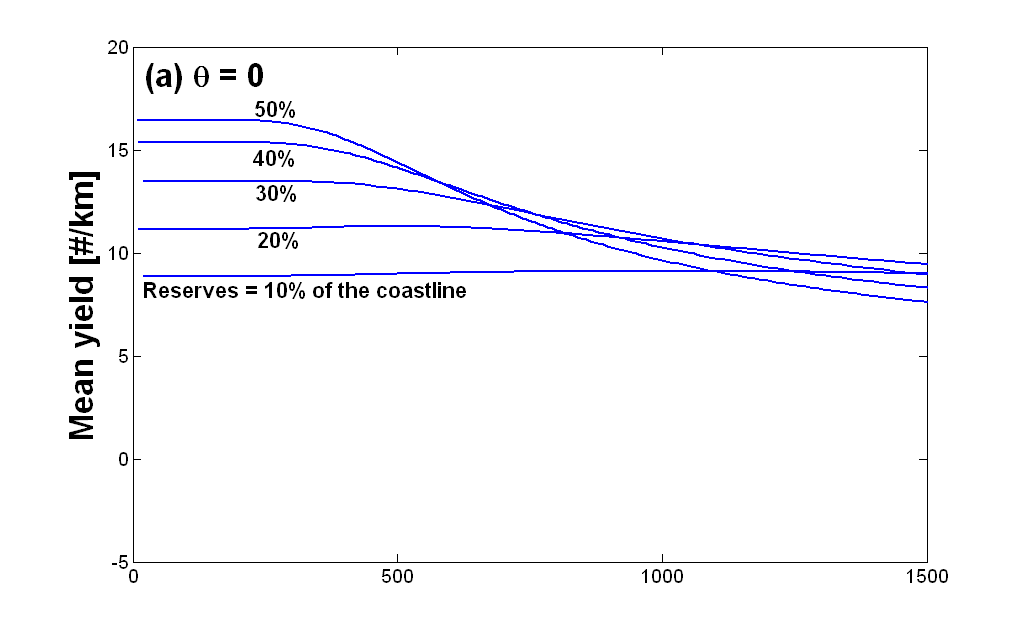

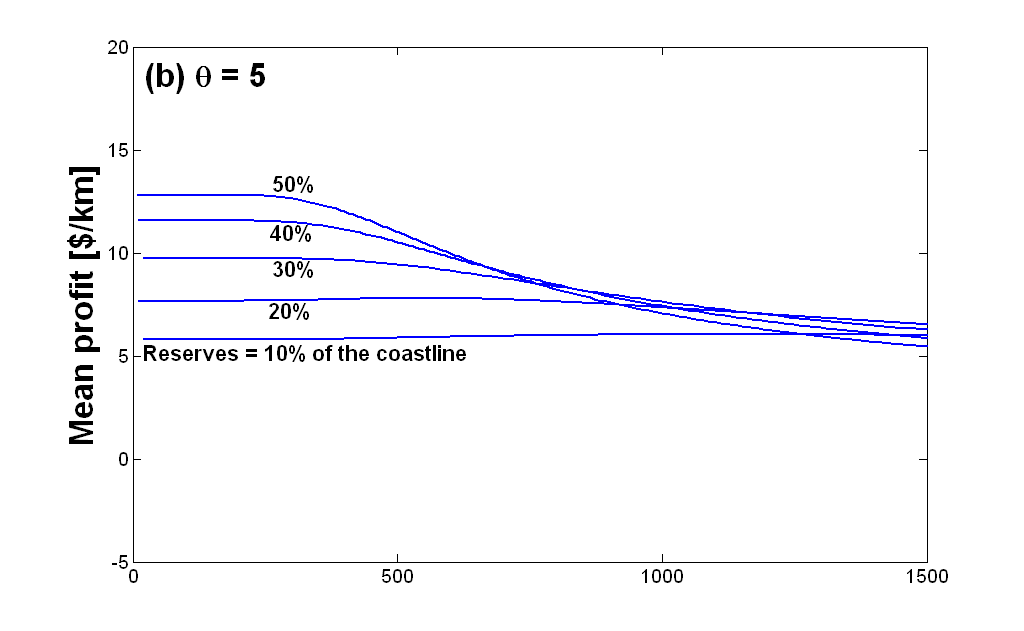

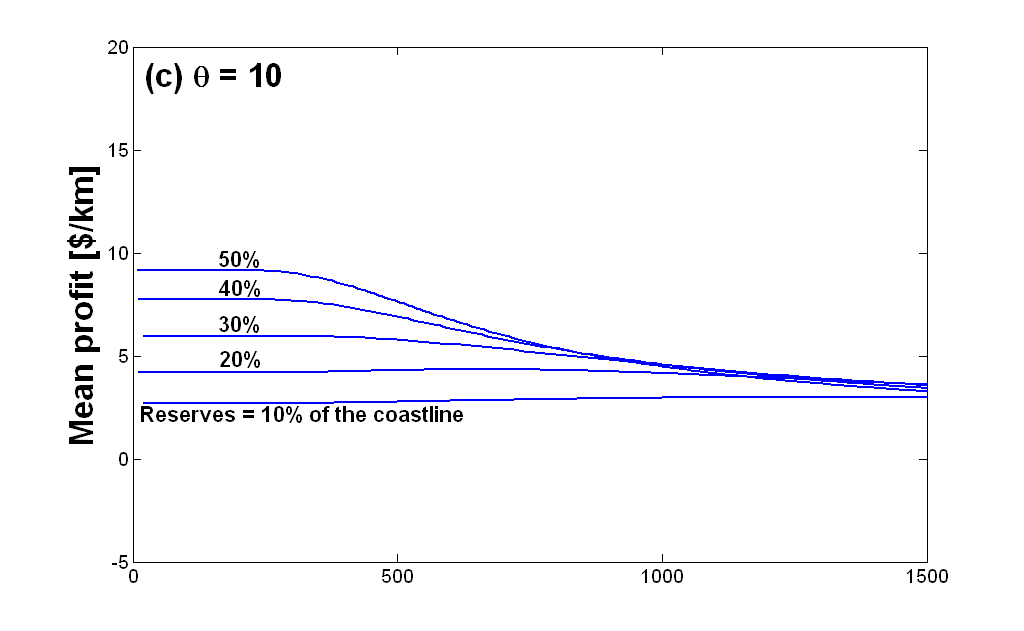

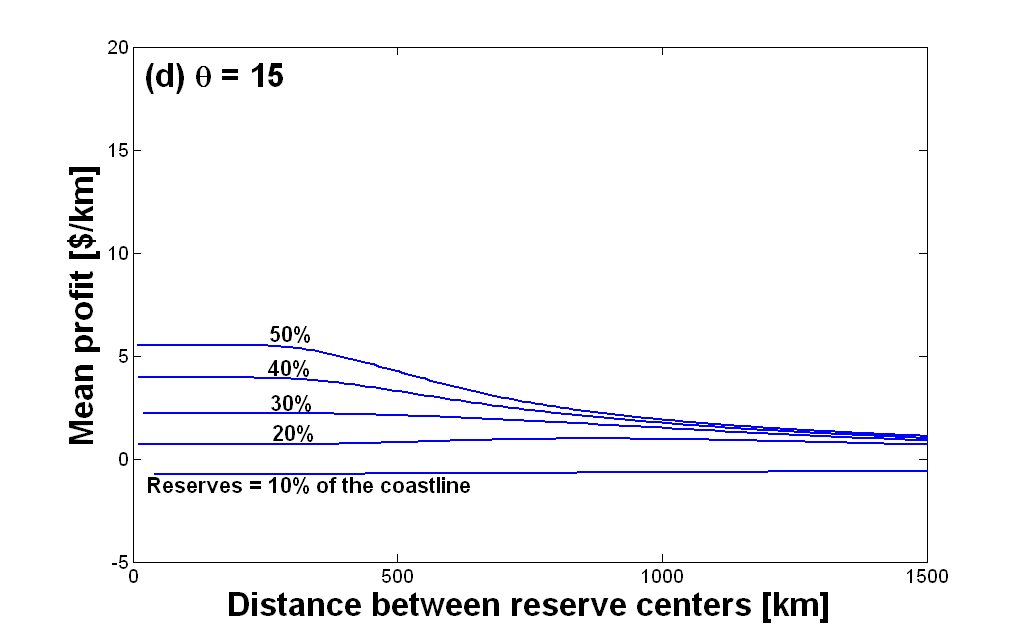


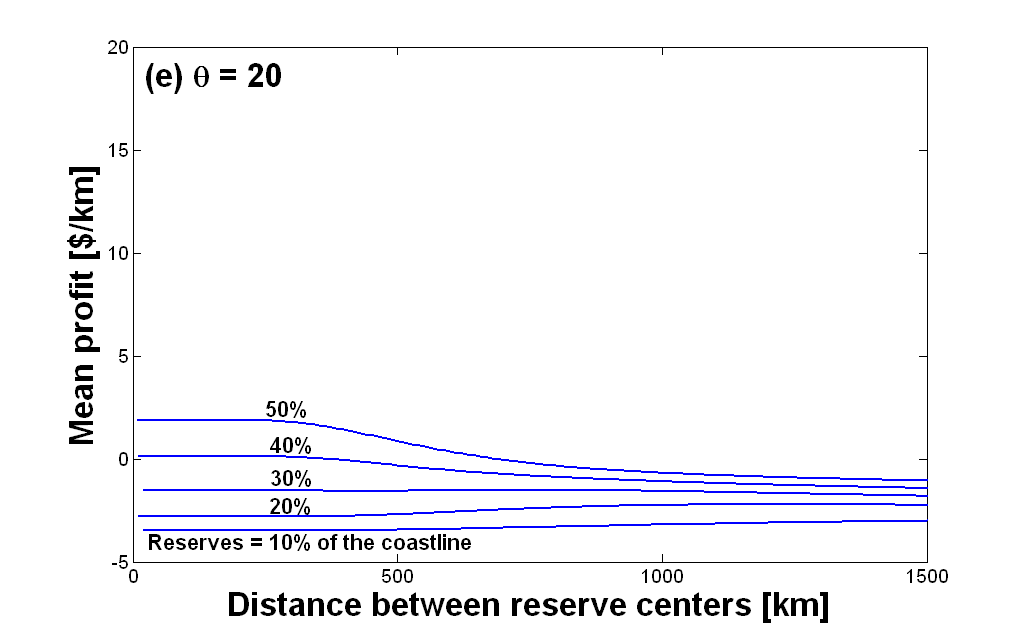


**Fig. S1.** Effect of reserve configuration on yield and profit, given escapement = 10%. Individual lines represent different proportions of the coast dedicated to reserves. Width of a single reserve equals the proportion of the coast dedicated to reserves multiplied by the distance between reserve centers (e.g., Given 50% of the coast in reserves, yield and profit is maximized when the distance between reserve centers is approximately equal to or less than 250 km, corresponding to an individual reserve width ≤ 125km). Mean larval dispersal distance = 100 km; results are qualitatively identical for smaller and larger mean dispersal distances (see Results). *M* = 0.1 and *P* = 1.0.

**Figure S2: Change in profit to fisheries operating under sub-optimal reserve-based versus optimal conventional management policies.**


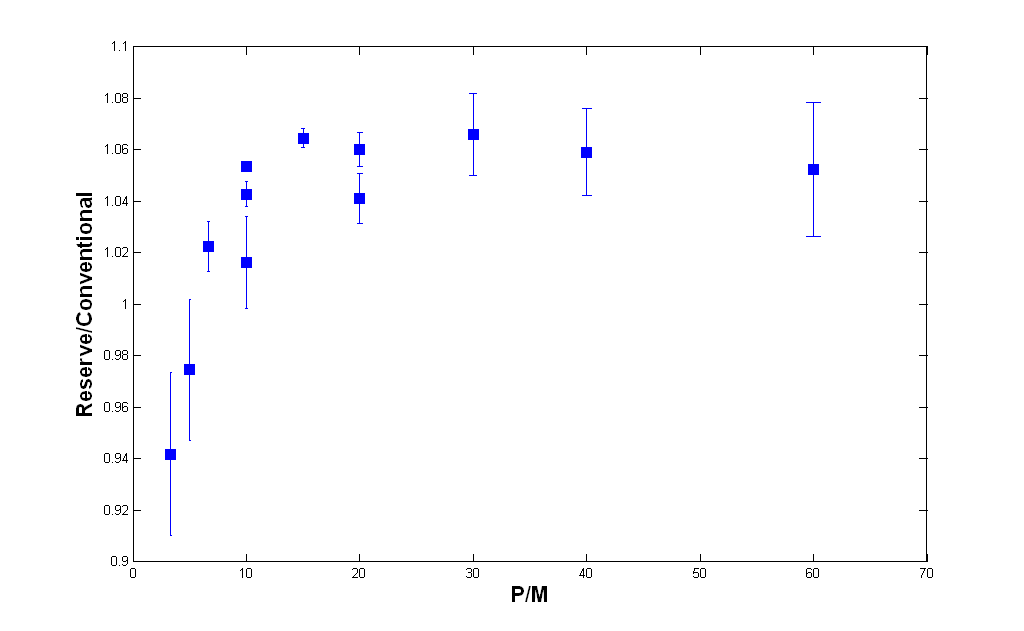

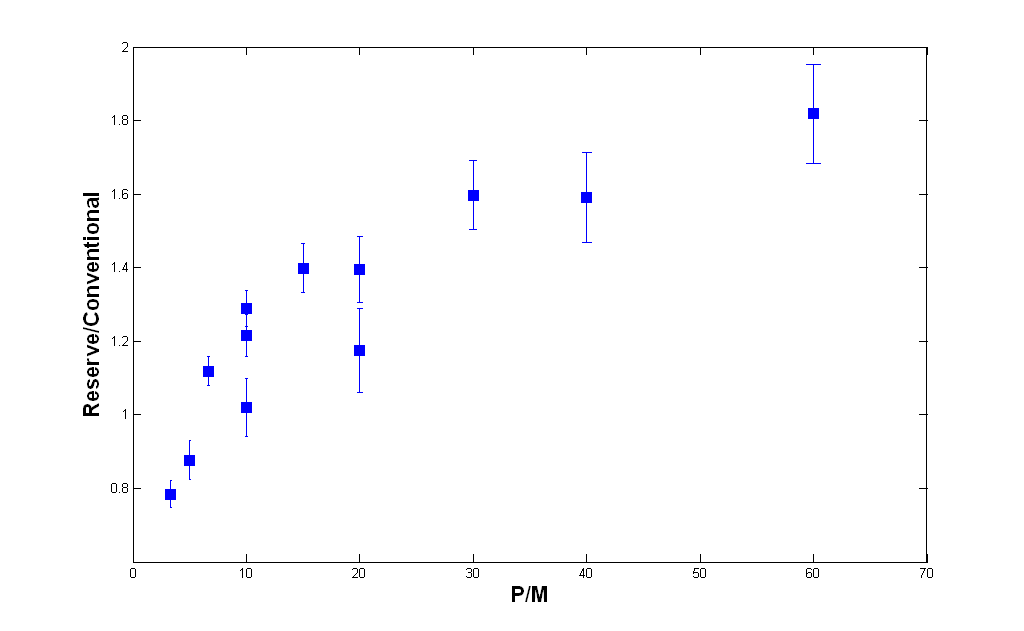


**(a)**

**(b)**

**Fig. S2.** Mean ± SE relative difference in profit to fisheries operating under sub-optimal reserve-based versus optimal conventional management policies. Data are plotted across the full factorial of productivity (*P*) and natural mortality (*M*) values in Table 1, and for each combination of demographic values profit statistics were calculated across *θ* = 5, 10, 15 and 20 stock effect scenarios. (a) Sub-optimal reserve-based management characterized by 20% of the coast dedicated to reserves and regulation across all species of a 35% escapement level. (b) Sub-optimal reserve-based management characterized by 60% of the coast in reserves and *θ*% escapement for each species. Note substantial increase in y-axis range. In both figures, the 2 scenarios resulting in a mean relative difference in profit less than one (i.e., on average, conventional management was more profitable) occur given *P* = 1 concurrent with *M* = 0.2 and 0.3.
